# Tomato 26S Proteasome subunit RPT4a regulates ToLCNDV transcription and activates hypersensitive response in tomato

**DOI:** 10.1038/srep27078

**Published:** 2016-06-01

**Authors:** Pranav Pankaj Sahu, Namisha Sharma, Swati Puranik, Supriya Chakraborty, Manoj Prasad

**Affiliations:** 1 National Institute of Plant Genome Research, Aruna Asaf Ali Marg, New Delhi-110067, India; 2 School of Life Sciences, Jawaharlal Nehru University, New Delhi-110067, India

## Abstract

Involvement of 26S proteasomal subunits in plant pathogen-interactions, and the roles of each subunit in independently modulating the activity of many intra- and inter-cellular regulators controlling physiological and defense responses of a plant were well reported. In this regard, we aimed to functionally characterize a *Solanum lycopersicum* 26S proteasomal subunit RPT4a (SlRPT4) gene, which was differentially expressed after *Tomato leaf curl New Delhi virus* (ToLCNDV) infection in tolerant cultivar H-88-78-1. Molecular analysis revealed that SlRPT4 protein has an active ATPase activity. SlRPT4 could specifically bind to the stem-loop structure of intergenic region (IR), present in both DNA-A and DNA-B molecule of the bipartite viral genome. Lack of secondary structure in replication-associated gene fragment prevented formation of DNA-protein complex suggesting that binding of SlRPT4 with DNA is secondary structure specific. Interestingly, binding of SlRPT4 to IR inhibited the function of RNA Pol-II and subsequently reduced the bi-directional transcription of ToLCNDV genome. Virus-induced gene silencing of SlRPT4 gene incited conversion of tolerant attributes of cultivar H-88-78-1 into susceptibility. Furthermore, transient overexpression of *SlRPT4* resulted in activation of programmed cell death and antioxidant enzymes system. Overall, present study highlights non-proteolytic function of SlRPT4 and their participation in defense pathway against virus infection in tomato.

Importance of the ubiquitin/26S proteasome (UPS) pathway in different plant-pathogen interactions is well recognized^1^,^2^,^3^,^4^,^5^. UPS pathway has been implicated in diverse aspects of eukaryotic cell regulation as it rapidly removes intracellular proteins^6^. In addition to these functions, it is also associated with immune responses to pathogen invasion. UPS components are indirectly or directly involved in signaling and regulation of non-host disease resistance, resistance gene-mediated responses, basal immunity and systemic acquired resistance^7^,^8^,^9^,^10^. It is used not only by the host cells in providing immunity and biotic stress responses, but also by pathogens, including viruses, for their own use^3^,^11^,^12^. Structurally, the 26S proteasome (26SP) in plants consists of a core particle (CP)/20S proteasome (20SP) and a regulatory particle (RP)/19S proteasome. The 20SP is involved in degradation of proteins whereas the 19S confers ATP- and Ub-dependence to the protease^13^. CP is a barrel-shaped ATP- and Ub-independent protease, built out of four stacked rings i.e., two inner and two outer. The inner rings consist of seven β subunits (β1to β7) while outer rings have seven α subunits (α1 to α7). These rings gate the access of proteins to the proteolytic chamber. The regulatory particle, on the other hand, is composed of two sub-complexes, the Lid and the Base. The Base contains six different RP Triple-A ATPases (RPTs) alongwith three RP Non-ATPase (RPN) subunits 1, 2 and 10. The RP Lid composed of eight RPNs (3, 5 to 9, 11 and 12). The RPTs unfold target proteins and open entrance of the 20SP chamber^14^,^15^. RPN subunits 1, 2 and 10 function as docking sites for different proteins.

Tomato leaf curl disease is caused by several strain/species of begomoviruses in India^16^,^17^, of which *Tomato leaf curl New Delhi virus* (ToLCNDV) is the most predominant and severe^16^. Due to lack of effective control measurements against the viruses, host resistance/tolerance is the main strategy for the efficient disease control. A few tomato (*Solanum lycopersicum*) cultivars/hybrids have displayed resistance/tolerance against the strains of tomato leaf curl virus in India^18^. Resistant sources to *Tomato leaf curl virus* are available in various accessions of *Solanum* species but the mechanism behind the resistance/tolerance has not been examined^19^,^20^. In our previous study, we identified a set of genes which were differentially expressed in ToLCNDV tolerant tomato cultivar H-88-78-1^21^. We reported higher abundance of UPS components like 26SP subunit RPT4 and Ubiquitin conjugating enzyme E2, along with many signaling and defense related genes, in tolerant tomato cultivar^21^. In the present study, we functionally characterized *Solanum lycopersicum* 26SP-RPT4a (SlRPT4) gene as a novel virus defense component of the tolerant cultivar. Here, we demonstrated that SlRPT4 protein may interfere with the ToLCNDV genome transcription and activates hypersensitive response (HR) in tomato.

## Results

### SlRPT4 has ATPase and DNA-binding activity

**Figure 1:**
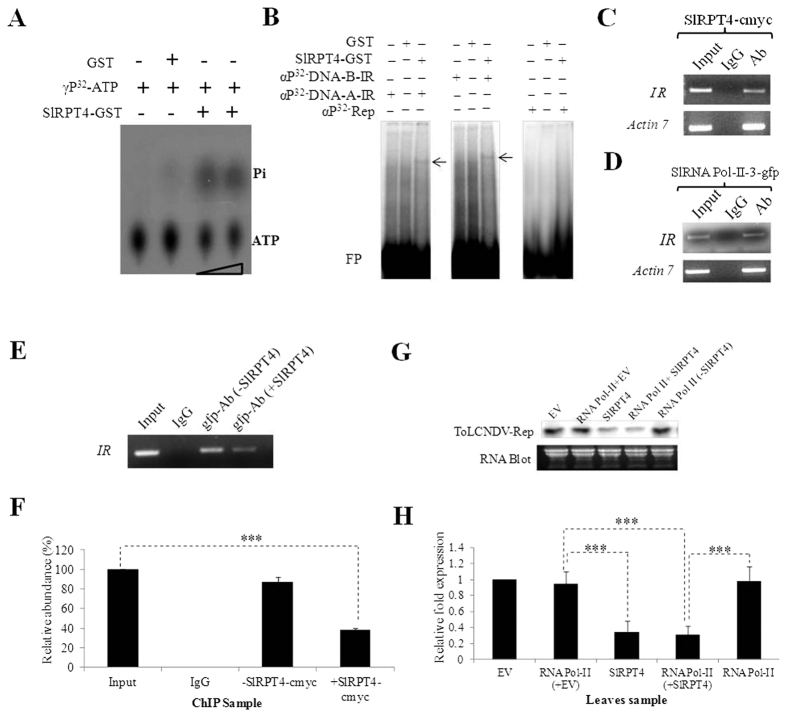
Molecular characterization of SlRPT4 protein. (**A**) Thin-layer chromatography (TLC) to evaluate ATPase activity. Figure shows dissociation of Pi from γP^32^-labelled ATP. Level of Pi was enriched upon increasing the amount of protein. GST protein was used as a negative control of the experiment. (**B**) DNA binding activity of SlRPT4-GST protein. Binding of SlRPT4 protein onto αP^32^-dCTP-labeled corresponding fragment of DNA-A-IR, DNA-B-IR and Rep regions are shown by retarded DNA-protein complex through EMSA on 6% native polyacrylamide gel. Signs, +/− represent the presence/absence of components. GST protein was used as a control substrate. *In vivo* binding assay was performed by transiently overexpressing SlRPT4 (**C**); SlRNA PolII subunit-3-gfp construct (**D**); and SlRPT4 and SlRNA PolII subunit-3 co-infiltration (**E**), in ToLCNDV infected leaves. Figures depict the amplification of IR fragments from the chomatin immuno-precipitated from the sample by using tag-corresponding to *c*-myc and gfp. (**E**) Relative abundance of IR specific fragments in the experimental samples. (**F**) Accumulation of *Tomato leaf curl New Delhi virus* ToLCNDV specific *Rep* transcripts in H^T^ (ToLCNDV infected cultivar H-88-78-1) and H^SlRPT4+T^ (*SlRPT4* silenced H-88-78-1 infected with ToLCNDV), (**G**) Northern hybridization showing the accumulation of Rep transcripts, (**H**) Relative accumulation of *Rep* transcripts in the leaf samples infiltrated with empty vector (EV), *SlRPT4*-myc and RNA Pol II-3-gfp construct alone, and co-infiltrated with RNA Pol II-3-gfp and SlRPT4-myc construct. Fragment corresponding to ToLCNDV-Rep gene was used as probe. Total RNA is shown as equivalent loading in the experiment. Data depicts means ± SD of three independent experiments (n = 3); *P < 0.05; **P < 0.01; ***P < 0.001.

To examine the biochemical properties of SlRPT4 protein, it was firstly purified as a SlRPT4-GST fusion protein (∼69 kDa) from E.coli strain BL21 (Supplementary Fig. 1). The protein showed ATP hydrolyzing activity in an ATPase assay suggesting that the protein can efficiently hydrolyze γP^32^-ATP and dissociate inorganic Phosphate (Pi) (Fig. 1A).

DNA binding activity of SlRPT4 protein was probed using amplified radiolabelled fragments of intergenic regions (IR) of DNA-A and DNA-B and with replication (Rep) regions by electrophoretic mobility shift assay (EMSA). After incubation, SlRPT4 protein formed complex only with DNA-A-IR and DNA-B-IR specific probes (Fig. 1B). However, this DNA-protein complex was not detected with the Rep-specific fragment, which lacks the secondary structure (Fig. 1B). This suggests that SlRPT4 protein may have binding affinity towards stem-loop structure of IR. Further, to check that the complex formation was not due to the binding affinity of GST protein, we incubated it with IR and Rep specific probes (Fig. 1B). Absence of any band showed that purified GST protein lacked DNA binding activity. Overall, the results suggested that SlRPT4 is an ATP-hydrolyzing and secondary structure specific DNA binding protein.

We also validated DNA-A-IR:SlRPT4 interaction through ChIP analysis (Fig. 1C,D). For this, SlRPT4-myc overexpression construct (i.e., pGWB17:SlRPT4-myc) was agro-infiltrated in ToLCNDV infected leaves of susceptible cultivar Punjab Chhuhara. After 3 day post-infiltration, cross-linked chromatin complexes were isolated. Fragmented chromatin was incubated with monoclonal anti-myc antibodies for immunoprecipitation of the complexes. DNA eluted was analyzed by PCR using primers corresponding to IR regions of ToLCNDV-DNA-A (Table 1). Result suggested that SlRPT4-myc has affinity to the DNA-A-IR of ToLCNDV (Fig. 1C).

We examined the possible interfering role of SlRPT4-IR complex with virus genome transcription. As RNA PolII plays an important role in the transcription of virus genome, gfp-tagged subunit-3 of *RNA PolII* was transiently expressed in ToLCNDV infected leaves. Immunoprecipitation was performed with the monoclonal anti-gfp antibody and obtained complexes were subjected to the PCR analysis. It was observed that DNA-A-IR specific primers were able to amplify the region from the immunoprecipitated samples, which suggested that the RNA PolII subunit binds to the IR region (Fig. 1D). Furthermore, both *SlRPT4* and *SlRNA PolII subunit-3* were transiently expressed into tomato leaves infected with ToLCNDV. Immunoprecipitation complex of DNA-A-IR:SlRNA PolII-3-gfp with anti-gfp antibodies were subjected to PCR analysis. It was revealed that the reproducibility of the IR-specific amplified product was significantly (*P* < 0.001) reduced ∼40% in the sample co-infiltrated with RNA PolII-3-gfp and SlRPT4-myc overexpression construct (Fig. 1E,F). However, tomato leaves co-infiltrated with RNA PolII-3-gfp and vector control showed relatively low reduction (∼15%) in the level of IR-specific fragments (Fig. 1E,F). These results suggested that SlRPT4 binds to the IR, which subsequently hinders the binding of RNA PolII complex, there by restricting ToLCNDV transcription in the infected cell.

To validate the ToLCNDV-specific transcript accumulation, *SlRNA PolII* over-expressed tissues with- and without-SlRPT4 co-expression were subjected to northern blot analysis. Tissues transiently expressing empty vector (EV) was used as experimental control. It was observed that upon *SlRPT4* expression, level of both complementary (Rep) and virus sense (CP) strand specific transcript were significantly (*P* < 0.001) down-regulated in comparison to co-expressed SlRNA PolII with EV tissues (Fig. 1G,H, Supplementary Fig. 2). Co-expression of *SlRNA Pol-II subunit* and *SlRPT4* also significantly (*P* < 0.001) reduces relative abundance of both Rep and CP transcript accumulation, in comparison to co-expressed *SlRNA PolII* with EV tissues (Fig. 1G,H, Supplementary Fig. 2). Rep-specific transcript accumulation on *SlRNA PolII* transiently expressed tissues with *SlRPT4* was significantly (*P* < 0.001) reduced, in comparison to only *SlRNA PolII* (without-SlRPT4) expressed tissues (Fig. 1G,H). Moreover, transient expression of *SlRNA PolII* in mock (EV) did not affect the level of Rep-specific transcript, as no significant differences between mock and *SlRNA PolII* overexpressed mock plant was observed (Fig. 1G). Virus sense strand specific-CP transcript accumulation was significantly (*P* < 0.001) down-regulated (∼80% reduction) in the tissues transiently co-expressed with *SlRNA PolII* and *SlRPT4* in comparison to *RNA Pol-II* alone (without-SlRPT4) infiltrated leaf tissues (Supplementary Fig. 2A,B). These results suggested that SlRPT4 may alter the level of ToLCNDV transcript in both directions, there by restricting virus infection in ToLCNDV infected tissues in tomato.

### Silencing of *SlRPT4* increases ToLCNDV induced symptom severity

Various studies have utilized the *Tobacco rattle virus* (TRV)-mediated VIGS system for the functional characterization of tomato genes^22^. Thus, we used TRV-VIGS vector to understand the role of *SlRPT4* in the alteration of ToLCNDV infection. The silencing efficiency of TRV-based VIGS system in cultivar H-88-78-1 was tested using an endogenous control *Phytoene desaturase* (*Slpds*). Tobacco was selected as reference to assess the degree of silencing (Supplementary Fig. 3). The characteristic photo-bleaching effect of PDS silencing started to appear at upper-most leaves at 10 days post-silencing and reached maximum at 21 days post-silencing (Supplementary Fig. 3). Transcript abundance of *Slpds* silenced plants were experimentally validated and showed >70% reduction in their accumulation, in comparison to mock-inoculated H-88-78-1 plant (Supplementary Fig. 3). Similar reduction in transcript level was recorded in the *Nbpds* silenced plants (Supplementary Fig. 3).

**Figure 2:**
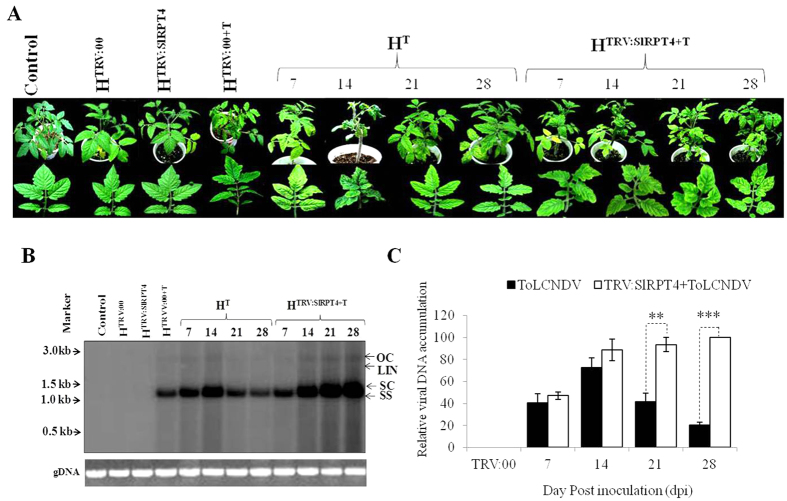
Plant phenotyping and molecular analysis of *SlRPT4* silenced tomato plants. (**A**) phenotype of experimental tomato plants. ToLCNDV tolerant cultivar H-88-78-1 plants were infiltrated with *Agrobacterium* containing the TRV:SlRPT4 construct (H^SlRPT4^). Subsequent to *SlRPT4* silencing process, ToLCNDV infection was performed. The photographs of the plants were taken at 7, 14, 21 and 28 days post inoculation (dpi). H^SlRPT4, SlRPT4^ silenced H-88-78-1; H^TRV:00^, vector infiltrated control plant; H^TRV:00+T^ vector infiltrated control plant infected with ToLCNDV; H^SlRPT4+T^, *SlRPT4* silenced H-88-78-1 infected with ToLCNDV; H^T^, cultivar H-88-78-1agroinfiltrated with ToLCNDV. Level of viral DNA in H^T^ (ToLCNDV infected cultivar H-88-78-1) and H^TRV:SlRPT4+T^ (*SlRPT4* silenced H-88-78-1 infected with ToLCNDV) at different dpi. (**B**) Southern blot of tomato genomic DNA from all experimental plants were hybridized with ToLCNDV-coat protein gene specific probe. Replicative forms of ToLCNDV genome are designate as open circular (OC), linear (Lin), supercoiled (SC) and single strand (SS). TRV:00 infiltrated H-88-78-1 was taken as a mock control. Ethidium bromide stained DNA from each experiments were shown as equivalent loading. (**C**) Relative accumulation of viral DNA in the samples H^T^ and H^SlRPT4+T^ at different time points. Data depicts means ± SD of three independent experiments (n = 3); *P < 0.05; **P < 0.01; ***P < 0.001.

For silencing of *SlRPT4*, a 231 bp fragment was cloned into the VIGS vector system and agro-infiltrated at two-leaf stage into cultivar H-88-78-1. ToLCNDV infection was performed subsequent to the silencing experiment. Tolerant cultivar H-88-78-1 challenged with ToLCNDV at two-leaf stage was used as an experimental control. RNA blot analysis revealed that the level of *SlRPT4* in silenced cultivar H-88-78-1 started declining 7 days post-silencing and reduced to minimum till 21 days post-silencing (Supplementary Fig. 4). A total of 75 plants from three independent experiments were tested for infectivity. Samples were denoted as H^TRV:00^ (vector infiltrated cultivar H-88-78-1 as Mock), H^TRV:SlRPT4^ (*SlRPT4* silenced cultivar H-88-78-1), mock infiltrated with ToLCNDV (H^TRV:00+T^), H^TRV:SlRPT4+T^ (*SlRPT4* silenced and ToLCNDV infected cultivar H-88-78-1) and H^T^ (ToLCNDV infected H-88-78-1). Infectivity scoring was performed at 21 dpi as described^21^ (Table 1). Leaf curl symptoms started to appear in H^TRV: SlRPT4+T^ within 7 dpi, whereas in H^T^, severe symptom initiation started at 14 dpi (Fig. 2A). Symptom remission started in upper systemic leaves after 14 dpi and at 21 dpi leaf-curling symptom was diminished in ToLCNDV-infected mock (H^TRV:00+T^) plants. H^TRV: SlRPT4+T^ plants failed to start symptom remission (Supplementary Fig. 5A,B). Severity of infection was higher in the H^TRV: SlRPT4+T^ and plants showed stunted growth, leaf curling and yellowing (Fig. 2A). Upon silencing, ∼70% plants showed the typical leaf curling symptom at 21 dpi. However, no contrasting phenotypic difference was observed in H^TRV:SlRPT4^ and H^TRV:00^ (mock) infiltrated plants.

Phenotypic changes in terms of viral infection and symptom development after down-regulation of SlRPT4 subunit, were validated by examining the accumulation of viral DNA-A at 7–28 dpi in both H^TRV:SlRPT4+T^ and H^T^ plants. Southern blot analysis revealed that viral DNA accumulation at 21 and 28 dpi was maximum (100%) in H^TRV:SlRPT4+T^, while H^T^ showed minimum (∼20%) accumulation at 28 dpi (Fig. 2B,C). Level of viral DNA accumulation was significantly higher in H^TRV:SlRPT4+T^ at 21dpi and 28 dpi (*P* < 0.01 and <0.001, respectively), in comparison to H^T^ plants. We also evaluated the accumulation of DNA-B upon *SlRPT4* silencing and found that level of corresponding molecule was significantly (*P* < 0.001) increased in *SlRPT4* silenced tomato cv. H-88-78-1 during ToLCNDV infection (Supplementary Fig. 6). Overall, the silencing of *SlRPT4* led to enhanced accumulation of both DNA-A and DNA-B, which signifies the involvement of this gene in providing tolerance against bipartite geminivirus.

### Transient overexpression of *SlRPT4* induces HR and Programmed cell death

**Figure 3:**
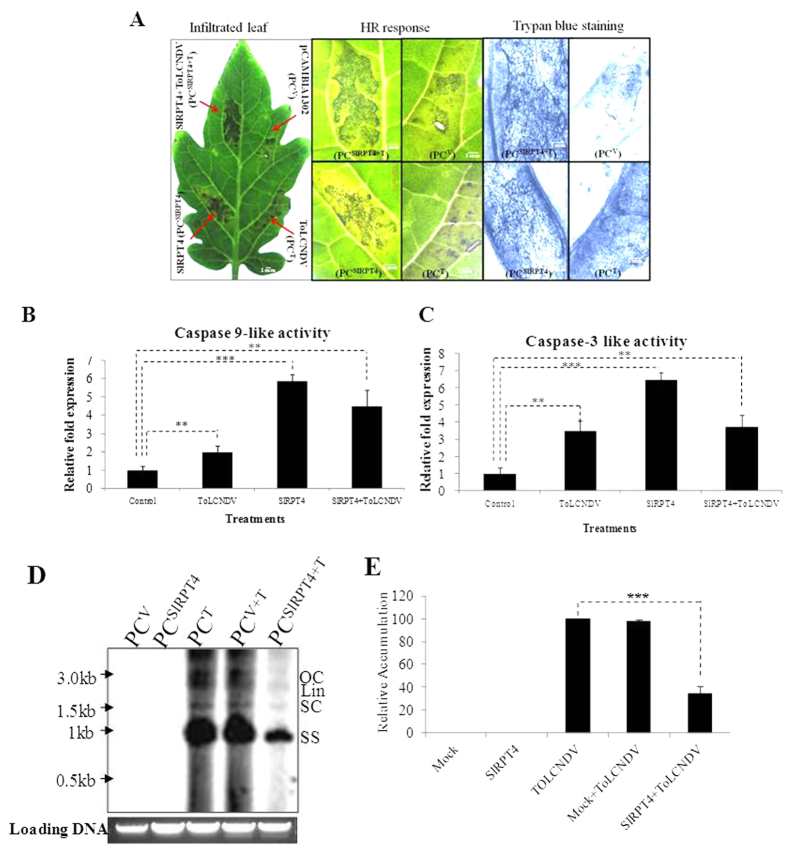
*Agrobacterium*-mediated transient overexpression of the *SlRPT4*. (**A**) Punjab Chhuhara leaves were agro-infiltrated with SlRPT4 overexpression construct (PC^SlRPT4^), pCAMBIA1302 vector (PC^V^), mixed treatment of SlRPT4 overexpression construct and ToLCNDV construct (PC^SlRPT4+T^), and ToLCNDV construct alone (PC^T^). Leaves were stained with trypan blue to confirm the cell death. Estimation of caspase 9-like (**B**) and caspase 3-like activity (**C**). Extracts from Punjab Chhuhara leaves of different treatments (Control, PC^V^; ToLCNDV, PC^T^; SlRPT4, PC^SlRPT 4^and SlRPT4+ToLCNDV, PC^SlRPT4+T^) were incubated with respective peptide substrates i.e., LEHD-AFC for caspase-9 like and DEVD-AFC for caspase-3 like activity, into caspase assay buffer. Their relative fluorescence was measured and enzymatic activities were normalized for protein concentration. (**D**) Accumulation of *Tomato leaf curl New Delhi virus* DNA in *SlRPT4* overexpressed tomato cultivar Punjab chhuhara. Southern blot of tomato genomic DNA from all experimental plants were hybridized with ToLCNDV-coat protein gene specific probe. (**E**) Relative accumulation of viral DNA in different treatments. Control, PC^V^; ToLCNDV, PC^T^; SlRPT4, PC^SlRPT4^ and SlRPT4+ToLCNDV, PC^SlRPT4+T^. Data depicts means ± SD of three independent experiments (n = 3); *P < 0.05; **P < 0.01; ***P < 0.001.

In order to investigate whether SlRPT4 gene is involved in programmed cell death (PCD) or not, *in planta* transient overexpression of *SlRPT4* was performed in a ToLCNDV susceptible tomato cultivar Punjab Chhuhara. Fully expanded leaves of cultivar were agro-infiltrated with overexpression construct *SlRPT4* (PC^SlRPT4^), ToLCNDV (PC^T^), mixed culture of ToLCNDV and overexpression construct SlRPT4 (PC^SlRPT4+T^). *Agrobacterium* containing the pCAMBIA1302 vector was infiltrated as a control measure and termed PC^V^. We found that overexpression of *SlRPT4* (PC^SlRPT4^) can produce HR symptoms as opposed to PC^V^ where less HR was observed (Fig. 3A). However, HR symptoms were also visible in infiltrated leaves of PC^T^ and PC^SlRPT4+T^ (Fig. 3A). HR-mediated programmed cell death (PCD) was examined by staining leaves with trypan blue reagent which showed characteristic cell death symptoms in PC^SlRPT4^ and PC^SlRPT4+T^ plants (Fig. 3A). However, leaf sample of PC^V^ also showed cell death symptom which may be due to the infiltration-associated compatible interaction with *Agrobacterium* containing the virulent vector. Interestingly, cell death symptoms were enriched upon ToLCNDV co-infiltration (Fig. 3A). In animal cells, during apoptosis cytochrome-c provokes a caspase-9 activating complex assembly, which consecutively triggers a cascade of caspases, including caspase-3^23^. Although, direct homologues of caspase genes are absent in the plant genome, involvement of certain caspase-like proteases in the control of cell death activation is reported in different pathogenic and non-pathogenic responses^24^,^25^. Therefore, synthetic fluorogenic substrates for caspase-9 (LEHD-AFC) and caspase-3 (DEVD-AFC) were used to detect caspase like activity in tomato cultivar Punjab Chhuhara with different treatments, mentioned previously. It was found that the caspase-9 and caspase-3 activity showed >5 fold induction in PC^SlRPT4^ in comparison to the control (Fig. 3B,C). PC^SlRPT4+T^ showed relatively lower accumulation of caspase 9- and caspase 3-like activity in comparison to the PC^SlRPT4^ (Fig. 3B,C). These observations suggested that up-regulation of *SlRPT4* could be involved in the regulation of PCD in plant cell.

We also studied viral DNA accumulation in the corresponding tissues through Southern blot analysis. Leaf tissues of PC^SlRPT4^, PC^T^, PC^SlRPT4+T^, PC^V^ and PC^V+T^ were harvested after 3 dpi, and subjected to the DNA isolation. It was observed that viral DNA accumulation was more in the case of PC^T^ in comparison to PC^SlRPT4+T^. Approximately 75% decrease in the viral DNA accumulation was observed in the *SlRPT4* overexpressed tissues, in comparison to PC^V^ and PC^V+T^ samples (Fig. 3D,E). This indicates that viral multiplication decreases due to activation of PCD by *SlRPT4* overexpression.

### Involvement of ROS in hypersensitive response

**Figure 4:**
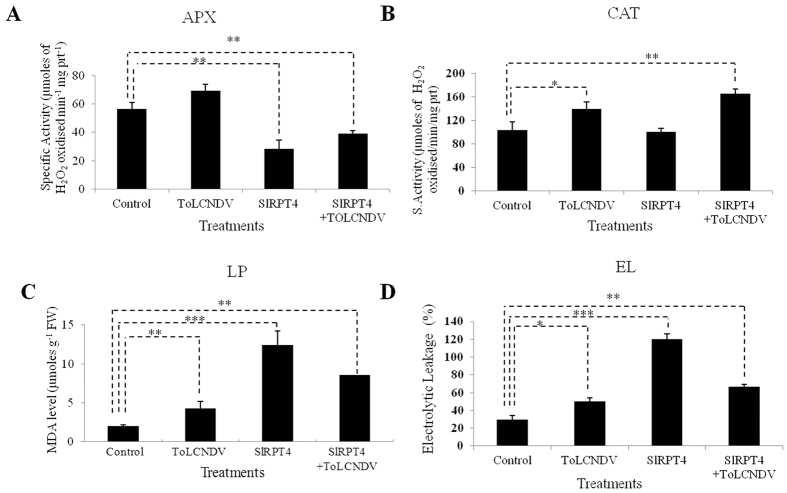
Estimation of antioxidant enzyme activity in cultivar Punjab Chhuhara. (**A**) Specific activity of APX was measured as 1 μ mol of ascorbate oxidized min^−1^. (**B**) Specific activity of CAT was measured as 1 μmol H_2_O_2_ oxidized min^−1^. (**C**) Levels of lipid peroxidation expressed in terms of MDA concentration. (**D**) Percentage electrolytic leakage. Data depicts means ± SD of three independent experiments (n = 3); *P < 0.05; **P < 0.01; ***P < 0.001. Control, PC^V^; ToLCNDV, PC^T^; SlRPT4, PC^SlRPT4^ and SlRPT4+ToLCNDV, PC^SlRPT4+T^.

The activity of enzymes like ascorbate peroxidase (APX) and catalase (CAT) were measured to test the production of reactive oxygen species (ROS) in the experimental tissues. In PC^SlRPT4^ treatment, a significant (*P* < 0.01) decrease in APX activity was observed in comparison to PC^V^ (Fig. 4A). However, specific activity of APX in PC^V^ was also significantly (*P* < 0.01) higher than PC ^SlRPT4+T^ (Fig. 4A). Similarly, level of CAT was significantly higher in PC^T^ and PC^SlRPT4+T^ in contrast to the PC^V^ (Fig. 4B). However, SlRPT4 transiently expressed tissue PC^SlRPT4^ showed non-significant change in CAT activity in comparison to PC^V^ tissues (Fig. 4B). In addition to the *SlRPT4* overexpressed Pujab chhuhara, antioxidant enzyme activities were also analyzed in SlRPT4-VIGS lines. Although, significant reduction in only APX activities was observed in H^TRV:00^ in comparison to H^TRV: SlRPT4^ (Supplementary Fig. 7A,B). Further, as a measure to assess membrane integrity, we evaluated the membrane damage and HR through Lipid peroxidation (LP) assay. The level of Malondialdehyde (MDA), which is an indicator of degree of LP, increased significantly (*P* < 0.001) in PC^SlRPT4^ with respect to vector control PC^V^ (Fig. 4C). LP was also significantly (*P* < 0.01) higher in PC^T^ infected leaves than PC^V^, however, MDA level decreased significantly (*P* < 0.01) in PC ^SlRPT4+T^ sample (Fig. 4C). With the aim to examine membrane damage as well as to validate the lipid peroxidation assay, we measured the electrolytic leakage (EL) in both *SlRPT*-transiently expressed and -silenced samples. It was observed that PC^SlRPT4^ exhibited significantly (*P* < 0.01) higher rate of ion leakage than the PC^V^ (Fig. 4D). Moreover, ToLCNDV infection (PC^T^) increased the rate of EL, which may be due to cultivar-specific response to the virus infection. Thus, membrane damage and ion leakage act as one of the marker elements of ROS in *SlRPT4*-mediated cell death. Interestingly, *SlRPT4* silenced samples showed non-significant difference in the level of both MDA and relative ion leakage in comparison to H^TRV:00^ tissues (Supplementary Fig. 7C,D).

## Discussion

Reports on the importance of 26SP subunits in independently targeting biological pathway regulators are increasingly apparent^26^,^27^,^28^. Most phenotypes of 26SP subunit mutants are defective in proteolysis function. Such mutants may also show defect in the function of individual subunits which necessitates re-evaluation of the role of each subunit. Apart from functioning in protein degradation and turnover, proteasomes have RNase activity. This activity appears to be an integral part of plant defense against viruses as RNase activity of 26SP subunits were highlighted during *in vitro* interaction with viral RNAs^29^,^30^,^31^. *Arabidopsis thaliana* proteasomal α5 subunit was identified as a factor to degrade *Tobacco mosaic virus* (TMV) and *Lettuce mosaic virus* (LMV)-derived RNAs *in vitro*^32^. Viral proteins may also interact and alter 20SP components catalytic activities. For example, HcPro of *Potato virus Y* (PVY) interacted with the α1, β2 and β5 subunits of the *Arabidopsis thaliana* 20SP and modulated the RNase activity leading to compromised defense response against these viruses^33^. It has also been suggested that the RNAse activity of 26S protesome subunits is differentially regulated through diverse extra-cellular signals^34^. Thus, proteasome-dependent RNase activity may represent an example of battle between the plant and pathogens, especially viruses.

The present study highlights novel function of a tomato 26SP subunit RPT4 (*SlRPT4*) in defense against geminivirus infection. It was found to contain an active ATPase domain (Fig. 1A) which makes it a potential protein to regulate various cellular and molecular functions. Proteins containing such AAA domain have been shown to involved in DNA replication, transcription control, degradation of protein, membrane fusion, microtubule regulation, signal transduction and the regulation of gene expression^35^,^36^. Majority of reports highlighting the specific function RPTs are established in yeast. For example, proteasomal ATPase RPT4 helps in the dislocation of endoplasmic reticulum-associated degradation (ERAD) substrates^37^. However very few reports are available, revealing the non-proteolytic functions of RPT4 in plants. A study showed that, rice RPT4 protein can interact with root architecture associated1 (RAA1) and facilitate the root growth and morphology^38^. In this regard, we further explored the non-proteolytic function of *SlRPT4* leading to defense against ToLCNDV.

Geminivirus genome contains an IR from which viral genes are transcribed in both the viral and complementary sense^39^. This bidirectional RNA polymerase II-type promoter is not only responsible for the transcription of viral genes but also contains specific sequence elements required for the viral DNA replication. SlRPT4 was found to possess DNA binding characteristics, more specifically to ToLCNDV-IR (Fig. 1B,C). The SlRPT4-IR complexes were observed when corresponding region of DNA-A and DNA-B were incubated with the SlRPT4-GST fusion protein. Interestingly, IR of both these components have hairpin loop structure, which strengthen our assumption that the binding of SlRPT4 with IR may be secondary structure specific. This structural specificity was also evaluated by SlRPT4 binding with the DNA-A specific Rep region (which does not possess any stem loop structure; Fig. 1B). Absence of any stem loop structure in Rep region of ToLCNDV prevented the specific protein-DNA complex formation, confirming that the binding of SlRPT4 with DNA is secondary structure specific for IR. Moreover, SlRPT4 inhibited the binding of RNA-Pol II onto IR (Fig. 1E,F) which resulted in altered transcription of ToLCNDV (Fig. 1G,H, Supplementary Fig. 2). Transient expression of *SlRPT4* significantly inhibited accumulation of Rep (complimentary sense strand) and coat protein (viral sense) gene specific transcripts (Fig. 1G,H, Supplementary Fig. 2) suggesting that SlRPT4 can inhibit bidirectional transcription of viral genes.

Our results establish a strong correlation between decrease in the level of ToLCNDV specific transcripts due to binding of SlRPT4 onto viral promoter. Thus, it can be posited that SlRPT4 has a non-proteolytic role in alteration of viral gene transcription through specific affinity with geminivirus-IR. As transcription and replication of viral genome are nuclear bound events, our study also suggests a novel nucleus associated function of SlRPT4 protein. Although, presence of 26SP complex in the nucleus has been reported in various studies, exact mechanism of transport is unclear^40^,^41^. Component of 26SP subunits are highly conserved but only RPT2a has been shown to have nuclear localization signal (NLS)^42^. Lack of NLS in SlRPT4 may denote that it has a NLS independent mechanism of transport into the nucleus.

This study also presents a direct evidence that inhibition of *SlRPT4* alters the tolerant characteristics of cultivar H-88-78-1 into susceptibility, both at phenotypic and molecular level (Fig. 2A–C, Supplementary Figs 5 and 6). VIGS of *SlRPT4* increases symptom severity in ToLCNDV inoculated tolerant cultivar H-88-78-1 (H^TRV:SlRPT4+T^; Fig. 2A). Increased titer of both viral DNA-A and DNA-B in H^TRV:SlRPT4+T^ can be associated with their higher replication as a consequential effect of *SlRPT4* silencing. Therefore, loss of *SlRPT4* function (i.e. the IR binding activity) assists in viral genome replication which is essential for viral infection and multiplication.

Transient overexpression of *SlRPT4* revealed that affected cells exhibited characteristic features of apoptotic cell death thereby restricting the ToLCNDV spread and infection (Fig. 3A–C). This proteasome-mediated cell death pathway is involved in caspase-9- and caspase-3-like activity (Fig. 3B,C). Several studies have reported that proteasomes are involved in conferring defense against pathogens^43^,^44^,^45^. Suppression of proteasome activity has been correlated with PCD in plants^23^,^46^. Contrastingly, diverse studies have revealed that activation of proteasome may also lead to PCD in plants^47^,^48^. For example, proteasome function was found to be essential for activation of PCD in heat stressed tobacco Bright-Yellow 2 cells^48^. During TMV infection in hot pepper, up-regulation of 26SP subunit RPN7 was associated with the activation of PCD^47^. Similar findings were also reported from animal systems, in which cell type dependent apoptosis was regulated by proteasome and lead to both initiation and inhibition of PCD^49^. Such evidences clearly suggest that the subunits of 26SP have complex function in PCD and precise role of each subunit needs to be investigated further. Our study highlights that upon transient expression of *SlRPT4*, transformed cells exhibited the characteristic cell death symptoms (Fig. 3A). We also showed that elevation of caspase-like enzymes possibly correlates with PCD. It is a localized response at the site of pathogen attack displaying PCD thereby limiting the spread of pathogens^50^. Thus, upon *SlRPT4* overexpression in a plant cell, activation of HR leads to PCD which in turn restricts the virus spread from the infected tissues into the subsequent cells. This suggest that SIRPT4a may have generic response in regulation of ROS mediated PCD (Although ToLCNDV is a non-necrotic virus).

In most cases of plant-pathogen interaction, reactive oxygen species (ROS) act as positive regulators of the HR and activate plant defenses^51^,^52^. Reduced ROS scavenging system (antioxidant) activities may also contribute to higher ROS accumulation leading to enhanced HR and defense against pathogens^53^,^54^,^55^. Hence, we also examined the level of ROS scavenging systems in both transiently expressed and VIGS-mediated silencing of SlRPT4 gene in susceptible and tolerant cultivar of tomato, respectively. Among the antioxidant enzymes, CAT and APX play important roles to cope-up excess ROS. Their down-regulation promotes increased ROS levels and cell death^56^,^57^. Examination of their activity showed a significant decrease in *SlRPT4*-silenced susceptible cultivar, PC^SlRPT4^ (Fig. 4A,B). In contrast, *SlRPT4*-silenced tolerant cultivar of tomato showed relatively higher expression of APX in comparison to the mock treated plants (Table S1). It suggested that increased level of APX in *SlRPT4*-silenced cultivar H-88-78-1 helps in detoxification of ROS. However, there was no significant increase in the level of CAT. In our previous study, we had constructed a tomato suppression subtractive hybridization library in between mock and ToLCNDV infected cultivar H-88-78-1^21^. Interestingly, it was observed that the transcripts similar to the catalase and their isoforms showed down-regulation and basal pattern of gene expression^21^. This data also support our finding that in ToLCNDV tolerant cultivar H-88-78-1 activation of ROS system helps in the reduction of viral spread. ROS can also produce lipid derivatives like conjugated dienes, hydroperoxides and malondialdehyde (MDA) by non-enzymatic oxygenation that directs membrane damage thus affecting cell viability^58^. Often, plant cell death is associated with high cellular electrolyte outflow^59^, which can be measured by ion leakage. Reduced cellular viability at the site of attack limits the spread of pathogens and may act as a source of signals for establishment of further defenses^50^,^60^. In this study, we found that MDA content was increased in *SlRPT4* transiently expressed plant along with the levels of relative ion leakage (Fig. 4C,D). Overall the above results suggest that ROS activation, altered response of antioxidant systems and reduced membrane stability may be related to the successful recognition of ToLCNDV infection and activation of defense signaling.

Collectively, these responses of SlRPT4 check the viral spread and thereby inhibit pathogenesis. It has an active ATPase domain which suggests it may have additional function; however, functional importance of ATP hydrolysis against virus infection in tomato needs to be explored further. SlRPT4 can interact with DNA, specifically at the stem loop structure, and can activate ROS and cell death process to regulate various defense related function against ToLCNDV. Up-regulation of SlRPT4 in tomato may help spreading ToLCNDV infection in two ways; firstly restricting ToLCNDV multiplication by activation of PCD and HR; secondly by binding with viral promoter to inhibit ToLCNDV transcription. However, exact mechanism of cell death by the SlRPT4 needs to be explored further. In this regard, protein-protein interaction study may also provide some evidence of SlRPT4 based activation of HR. We are currently developing a stable transgenic line in tomato, to further investigate the role of SlRPT4 in PCD. The emerging picture suggests that 26SP mediated defense occurs at different levels of plant-virus interaction. In addition, demonstrated novel antiviral role of RPT4 will promote plant biologist and virologist to functionally characterize other components of the UPS for better understanding of plants and pathogens in incompatible interactions.

## Materials and Methods

### Plant material and growth conditions

*Solanum lycopersicum* (cultivar H-88-78-1 and cultivar Punjab Chhuhara) seeds were sown in composite soil (peat compost to vermiculite, 3:1 v/v), germinated and maintained in a growth chamber containing 2 cabinets (PGC-6L; Percival Scientific Inc., USA) under 14 h light/10 h dark cycle at 25 °C, 70% relative humidity, and a light intensity of approximately 250 *μ*mol photons m^−2^ s^−1^. For silencing experiment, the temperature was maintained at 22 °C throughout as higher temperature inhibits the T-DNA transfer.

### Recombinant protein expression and purification

For bacterial expression, pGEX4T2:SlRPT4 recombinant construct was prepared through specific primers listed in Table S1. GST-tagged-SlRPT4-protein was expressed in BL21 (DE3) strain. SlRPT4-GST fusion protein was induced by adding 1 mM isopropyl *β*-D-thiogalactopyranoside (IPTG) fallowed by re-suspension in lysis buffer [10 mM PBS, pH 7.0; 1 mM phenylmethylsulfonyl fluoride (PMSF), 2 μl/ml protease inhibitors] and sonication. After centrifugation for 10 min at 12000 rpm, obtained pellet was dissolved in IBS buffer (G-biosciences) at 4 °C for 1 h. After centrifugation, supernatant was purified by affinity chromatography using Glutathione-Sepharose 4B. SlRPT4-GST fusion proteins were eluted through treatment with elution buffer (20 mM reduced Glutathione) and further dialyzed with dialysis buffer [50 mM 4-(2-hydroxyethyl)-1-piperazineethanesulfonic acid (HEPES), pH 7.5; 40 mM KCl].

### ATPase assay

Thin-layer chromatography (TLC) method was used for ATPase assay. Briefly, different amounts of protein were incubated in ATPase buffer [10 mM Tris-HCl (pH 8.0), 5 mM MgCl_2_, 50 mM KCl, 10 mM DTT, and 100 μg/ml BSA] containing 0.2 μCi of γP^32^ labelled-ATP (Perkin Elmer Life Sciences, USA), at 37 °C for 30 minutes. TLC was performed. Reaction sample was spotted on a polyethyleneimine TLC plate. Air dried reaction mix was as resolved on a solvent consist of 0.5 M Lithium chloride and 1 M Formic acid. TLC paper was air dried and subsequently exposed to phosphor-screen and subjected to image scanning using phosphor-imager (Typhoon 9210).

### Electrophoretic mobility shift assay (EMSA)

EMSA was performed according to the protocol described^61^. PCR amplified product of DNA-A-IR, DNA-B-IR and Replication associated gene fragments were labeled with αP^32^-dCTP at 5′ end using Klenow fragment of DNA polymerase I. Further, binding reaction between purified SlRPT4-GST fusion protein and αP^32^-dCTP labeled IR fragment was performed in 30 μL of binding buffer [75 mM HEPES, pH 7.5; 150 mM KCl, 0.1 mM DTT, 1 mM EDTA, pH 8; 5 mM MgCl_2_, 30% (w/v) glycerol and 0.1 mg/ml BSA] containing poly (di-dC). Samples were incubated at 25 °C for 30 min. After incubation samples were resolved in 10% PAGE containing 2% glycerol in 1X-TAE (40 mM Tris base, 20 mM Acetic acid and 1 mM EDTA, pH-8.0) buffer at 10 mA. After running, the dried gel were exposed to phosphor-screen and subjected to image scanning using phosphor-imager (Typhoon 9210).

### Chromatin immunoprecipitation assay (ChIP)

To examine the DNA binding affinity of SlRPT4 with region corresponding IR of ToLCNDV DNA-A, ChIP assay was performed according to the protocol described^62^. Briefly, the process implicated were immune-precipitation, reverse cross-linking, digestion of protein followed by DNA precipitation. For this, leaves of cultivar Punjab Chhuhara were agro-infiltrated with pGWB17:SlRPT4-cmyc construct. Approximately, two gram of agroinfiltrated sample was subjected to cross-linking with the buffer containing (0.4 M Sucrose, 10 mM Tris-Cl (pH-8), 1 mM PMSF, 1 mM EDTA, and 1% Formaldehyde). Submerged leaves were vacuum-infiltrated for 20 minutes followed by 10 minutes vacuum infiltration of 2 M Glycine to stop cross-linking. Downstream processing including immune-precipitation, reverse cross-linking, digestion of protein followed by DNA precipitation was performed as described^49^. Tissues were grounded to the fine powder using liquid Nitrogen and re-suspended and homogenized into Nuclei isolation buffer (0.25 M Sucrose, 10 mM Tris-HCl pH-8, 10 mM MgCl_2_, 1% Triton X-100, 5 mM β-mercaptoethanol, 1 mM PMSF, Protease inhibitor). Homogenized mix was filtered through muslin cloth and centrifuged at 11,000 *g* for 20 min at 4 °C. Resultant pallet was dissolved in nuclei lysis buffer. This mix was sonicated by 8 times at 60% for 15 sec followed by centrifugation at 13000 *g* for 10 min at 4 °C. Sonicated chromatins were subjected to immuno-precipitation via anti-cmyc and anti-H_3_K_4_Me_3_ antibodies to obtain the desired genomic DNA fragments of ToLCNDV-IR and *Actin7*, respectively. As the negative control anti-IgG-antibody immunoprecipitation was used, while input control was non-immunoprecipitated sample. Obtained DNA was used for PCR amplification of targeted region of IR and *Actin7* by region specific primers (Table S1). Amplified products were resolved in 1% agarose gel.

### TRV-mediated VIGS and agroinfiltration

TRV mediated gene silencing was performed using the pTRV1 and pTRV2 vector (kindly provided by Dinesh-Kumar, Plant Biology Department, University of California, USA). The target gene silencing construct were prepared using pTRV1 and pTRV2 vectors according to the established protocol^63^. Silencing efficiency of TRV-based VIGS system in cultivar H-88-78-1 was evaluated by silencing of an endogenous control *Phytoene desaturase* (SGN-U593894) along with *Nicotiana benthamiana* (to assess the degree of silencing). Tomato *26S proteasomal subunit RPT4a* (SGN-U566414) which was reported to differentially expressed in response to ToLCNDV infection in tolerant cultivar H-88-78-1, was selected for the silencing study. To minimize the effect of off target silencing, 231 bp fragment from 3′-UTR of *SlRPT4* was PCR amplified from tomato cDNA using primer (Table S1) and cloned into pTRV2 between *Xho*I/*Bam*HI sites to form pTRV2:RPT4. The constructs were introduced into *Agrobacterium* strain EHA105 and cells harboring pTRV1 and pTRV2 or pTRV derivatives were cultured in liquid broth (containing 100 μm MES buffer; pH 5.5) supplemented with antibiotics (50 μg ml^−1^ Kanamycin and 50 μg ml^−1^ Rifampicin) and grown overnight at 28 °C. These cultures were centrifuged at 1,000× g for 10 min and re-suspended in the same volume of re-suspension buffer (10 mM MgCl_2_, 100 μM Acetosyringone and 1 mM MES buffer; pH 5.5). After adjusting OD_600_ at 1, transformed cells were incubated at room temperature for 3 h. These cultures were mixed according to the experimental set up (i.e. control, mock and gene silencing constructs) in 1:1 ratio and infiltrated at two leaf stage into tomato leaves by pressing a 1 ml syringe against the lower surface. Subsequent to silencing experiment ToLCNDV agro-infection was done by piercing with a needle at stem node. Tomato cultivar H-88-78-1 was termed on the basis of various treatments, namely H^TRV:00^ (TRV:00 vector infiltrated H-88-78-1 as Mock), H^TRV:SlRPT4^ (*SlRPT4* silenced cultivar H-88-78-1), H^TRV:SlRPT4+T^ (*SlRPT4* silenced cultivar H-88-78-1 and ToLCNDV infected), H^TRV:00+T^ (Mock plants infected with ToLCNDV) and H^T^ (only ToLCNDV infected H-88-78-1). Each experiment was repeated three times.

### Agrobacterium-Mediated Transient Overexpression of *SlRPT4*

For Agrobacterium-mediated transient overexpression, full length cDNA of SlRPT4 gene was amplified using primer pairs (Table S1) and cloned into pCAMBIA1302 vector between *Nco*I/*Spe*I restriction sites. The resulting construct was transformed into *A. tumefaciens* strain GV3101. Transformed cells carrying pCAMBIA1302:SlRPT4 along with the empty vector were cultured and re-suspended as described above. The re-suspended culture’s absorbance (OD_600_) was adjusted to 0.8 and infiltrated into tomato leaves at two leaf stage. Tomato cultivar Punjab Chhuhara inoculated with different constructs were named as PC^V^ (pCAMBIA1302 vector inoculated cultivar Punjab Chhuhara), PC^SlRPT4^ (*SlRPT4* overexpressed cultivar Punjab Chhuhara), PC^SlRPT4+T^ (*SlRPT4* overexpressed cultivar Punjab Chhuhara and ToLCNDV infected) and PC^T^ (only ToLCNDV infected cultivar Punjab Chhuhara). After 2 to 3 days, the infiltrated leaves were used for molecular analysis analysis.

### Southern hybridization

Total DNA was isolated from leaves of each infiltrated plant by the cetyl-trimethylammonium bromide (CTAB) method^64^. For Southern hybridization, equal amount of total DNA (5 μg) from each experimental samples were electrophoresed on 1% agarose gel in TBE [Tris-borate EDTA; 45 mM Tris-borate, 1 mM EDTA (pH 8)] and transferred to positively charged nylon membrane (HYBOND-N^+^, Amersham Bioscience, USA). ToLCNDV-specific CP gene (DNA-A) and BC1 (DNA-B) probes were prepared as described^20^ and DNA fragments were labeled with [α^32^P]dCTP using NEBlot Kit according to the manufacturer’s protocol (New England Biolabs).

### RNA blot analysis

Total RNA was isolated using TRI reagent (Sigma-Aldrich, USA) from tomato leaves subjected to different treatments. For Northern blot analysis, 10 μg total RNA was electrophoresed on 1.2% denaturing formaldehyde agarose gel in 1X MOPS running buffer and transferred to positively charged nylon membrane (Hybond-N^+^, Amersham Bioscience, USA). For the viral replication specific transcript accumulation, Rep and CP gene of ToLCNDV were PCR amplified using specific primer pairs for probe preparation (Table-S1). Similarly probes specific to *Slpd*s, *Nbpds* and *SlRPT4* were amplified using gene specific primers listed in Table S1. Probes were labeled with [α^32^P] dCTP using NEBlot Kit according to the manufacturer’s protocol (New England Biolabs). Phosphor-imager (Typhoon-9210, GE Healthcare, USA) was used to scan the blots and further densitometry was done through available software (Quantity One; Bio-Rad, USA).

### Trypan blue staining

Trypan blue staining was performed according to protocol^65^. In brief, cell death in the tomato leaves was examined by staining with trypan blue reagent (10 ml Lactic acid, 10 ml Glycerol, 10 g phenol, and 10 mg Trypan blue). The treated leaves were boiled for 1 min in capped falcon tube and distained overnight in Chloral hydrate. Photographs were taken using a stereomicroscope (SMZ 1500, Nikon, USA).

### Measurement of Caspase-like activity in SlRPT4 overexpressed tomato

Leaves from different treatments were ground and homogenized in caspase lysis buffer (G-biosciences, St Louis, MO, USA). Samples were incubated in ice for 15 min, further centrifuged, and supernatants collected. Resultant supernatants (50 μl) were mixed with 50 μl of 2X caspase assay buffer with 150 μM LEHD-AFC (G-biosciences, St Louis, MO, USA) for caspase 9-like activity and 150 μM DEVD-AFC (G-biosciences, St Louis, MO, USA) for caspase 3-like activity, as peptide substrates. Fluorescence generated through the AFC hydrolysis was quantified by spectrofluoro photometer (Varian, Victoria, Australia) at 400-nm excitation and 505-nm emission wavelengths after incubation at 37 °C for 60 min. To evaluate the protein concentration, enzymatic activity was normalized with the fold activity of control extracts. Three independent experiments were conducted for measurement of fold activity.

### Measurement of catalase (CAT) and ascorbate peroxidase (APX) activity

CAT activity was assayed according to protocol^66^. For this, 100 mg of leaves from each set of treatments were ground in liquid nitrogen, and suspended in 100 mM potassium phosphate buffer (pH 7.0) with 1 mM EDTA (pH 8.0). After centrifugation at 4 °C, supernatants were taken and measured for CAT activity with 60 mM H_2_O_2_ at 240 nm wave length using a UV-visible spectrophotometer (UV2550, Shimadzu, Japan). For APX, leaves were homogenized in the homogenization buffer (50 mM HEPES; pH 7.0, 0.1 mM EDTA; pH 8.0). After centrifugation at 4 °C, supernatants were measured for the APX activity with 0.03 mM Ascorbate and 0.1 mM H_2_O_2_ at 290 nm wavelength. Protein estimation was carried out using BSA as standard^67^.

### Lipid peroxidase (LP) activity and electrolytic leakage (EL)

The lipid peroxidation levels in the experimental samples were evaluated through determining Malondialdehyde (MDA) content by 2-Thiobarbituric acid (TBA) reaction^68^. For this 100 mg tissues were homogenized in 10 mM sodium phosphate buffer (pH 7.4) followed by centrifugation at 4,000 g for 5 min at room temperature. The supernatant (100 μl) was added to a reaction mixture containing 8.1% SDS (w/v), 20% Acetic acid (pH 3.5) (w/v) and 0.8% aqueous TBA (w/v). These mixtures were incubated at 98 °C for 1 h, cooled to room temperature and centrifuged at 4,000 g for 5 min. Incubated samples were subjected to the measurement of absorbance and non-specific absorbance at 535 nm and at 600 nm wavelength, respectively.

Electrolytic leakage (EL) was assessed according to protocol described elsewhere^69^. After *Agrobacterium* infiltration, the infiltrated leaf from the different treatments were collected and analyzed. Fifteen leaf discs (7 mm in diameter) were floated on the 0.4 M sorbitol. The leaf discs were incubated in the dark for 12 h and the initial conductivity (E1) was recorded using a microprocessor based conductivity meter (Model 1601, ESICO, India). The pre-incubated leaves were boiled for 5 min, cooled to room temperature and further subjected to measure final conductivity (E2). Percentage EL was calculated using the formula i.e., (E1/E2) × 100.

### Statistical analysis

Experimental data represent means of three independent tests. The significance (at *P < 0.05, **P < 0.01, ***P < 0.001) differences between mean values of control and each treatment samples were statistically carried out via graphpad *t* test calculator software (http://graphpad.com/quickcalcs/ttest1.cfm).

## Additional Information

**How to cite this article**: Sahu, P. P. *et al.* Tomato 26S Proteasome subunit RPT4a regulates ToLCNDV transcription and activates hypersensitive response in tomato. *Sci. Rep.* **6**, 27078; doi: 10.1038/srep27078 (2016).

## Supplementary Material

Supplementary Information

## Figures and Tables

**Table 1:** Infectivity scoring of *Tomato leaf curl New Delhi virus-*infiltrated control, mock and *SlRPT4* silenced plant.

Plant	Plant infected/inoculated	Severe symptom appearance	Symptom severity^a^	Over all grade^b^
H-88-78-1 (H^T^)	5/75	14	+	T
Mock (H^TRV:00+T^)	7/75	13	+	T
SlRPT4 silenced (H^TRV:SlRPT4+T^)	51/75	7	++++	HS
Mock (H^TRV:00^)	–	–	–	–
SlRPT4 silenced (H^TRV:SlRPT4^)	–	–	–	–

Infectivity indexing was done on systemic leaves of experimental plants at 21 days post-inoculation, following the protocol described elsewhere^21^. Briefly, a) +, Least severe; ++, moderately severe; +++, severe; ++++, highly severe. b) T, Tolerant (1–20%); MT, Moderate tolerant (20.1–40%); S, Susceptible (40.1–60%); HS, Highly susceptible (60.1–100%). ‘–’ represent non-ToLCNDV infected samples.
