# Supplementary material for: Tomato 26S Proteasome subunit RPT4a regulates ToLCNDV transcription and activates hypersensitive response in tomato
Source: Sci Rep. 2016 Jun 1;6:27078. doi: 10.1038/srep27078 (PMC4890432; doi:10.1038/srep27078)
Supplement: Supplementary Information [file srep27078-s1.pdf]

Tomato 26S Proteasome subunit RPT4a regulates ToLCNDV transcription and activates hypersensitive response in tomato

Pranav Pankaj Sahu[1,2], Namisha Sharma[1], Swati Puranik[1], Supriya Chakraborty[2], Manoj Prasad[1*]

[1] National Institute of Plant Genome Research, Aruna Asaf Ali Marg, New Delhi-110067, India

[2]School of Life Sciences, Jawaharlal Nehru University, New Delhi-110067, India

[*] **Corresponding author:** Manoj Prasad, E-mail: manoj_prasad@nipgr.res.in

National Institute of Plant Genome Research (NIPGR), Aruna Asaf Ali Marg, New Delhi-110 067, India, Tel: ++91-11-26735160, Fax: ++91-11-26741658

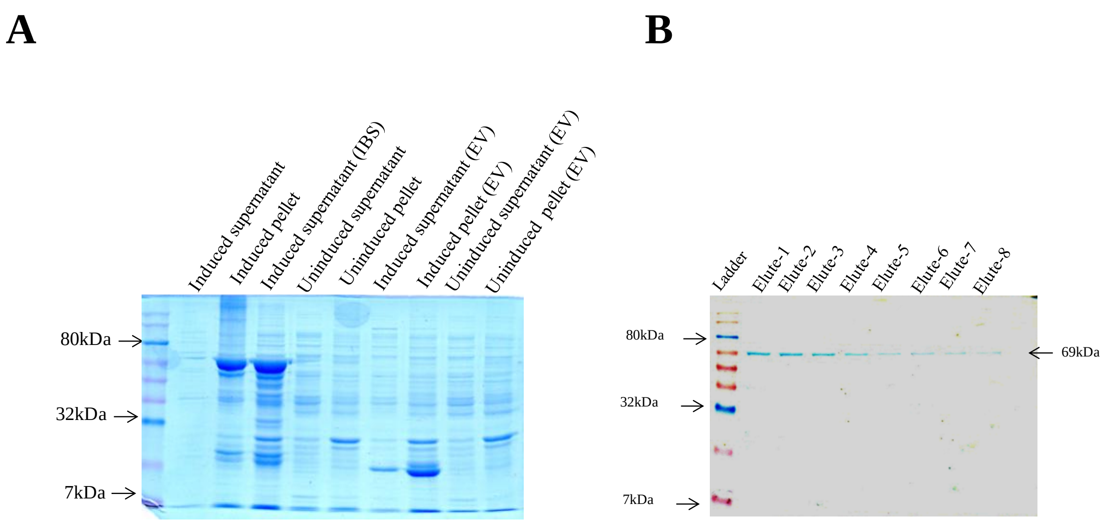

Supplementary Figure 1. Bacterial expression and purification of SlRPT4a. (A) Coomassie brilliant blue stained 12% SDS-PAGE containing induced and uninduced SlRPT4-GST fusion protein (69kDa); (B) Purified SlRPT4 protein. Band represents fractions of GST-affinity purification of SlRPT4-GST fusion protein.

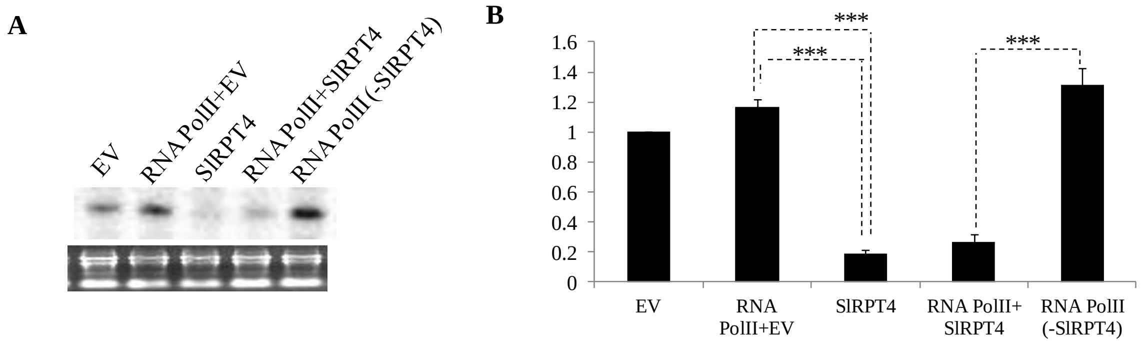

Supplementary Figure 2. Accumulation of Coat protein (CP) specific transcripts, (A) Northern hybridization showing the Relative accumulation of CP transcripts in the leaf samples infiltrated with empty vector (EV), SlRPT4-cmyc and RNA Pol II-3-gfp construct alone, and co-infiltrated with RNA Pol II-3-gfp and SlRPT4-muc construct. Fragment corresponding to ToLCNDV-CP gene was used as probe. Total RNA is shown as equivalent loading in the experiment. Data depicts means±SD of three independent experiments (n=3); *, $P<0.05$; **, $P<0.01$; ***, $P<0.001$.

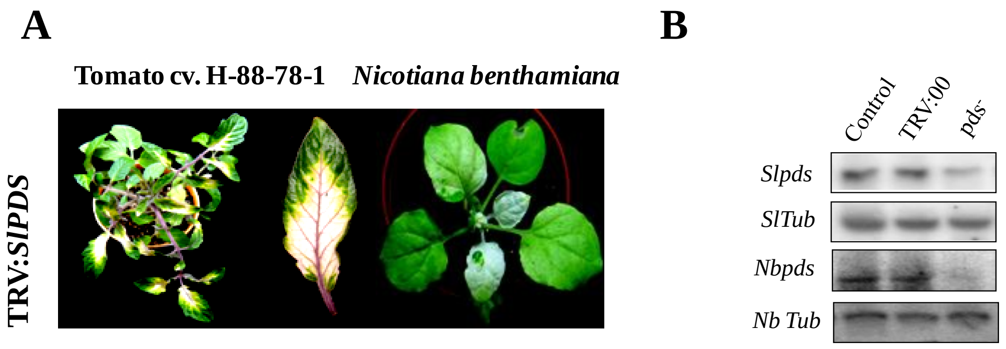

Supplementary Figure 3. TRV-based VIGS in tomato and *Nicotiana benthamiana*. (A) Phenotype of tomato and *N benthamiana* plant at 21 day post-silencing. Fragment corresponding to *Slpds* and *Nbpds* was used to produce TRV-based gene silencing construct. After 21 day post silencing typical leaf bleaching symptoms was observed. (B), Northern blot analysis to evaluate the relative level of pds gene in control, vector infiltrated (TRV:00) and *pds* silenced (pds$^-$) plants. Tubulin gene from tomato and *Nicotiana* were used as internal control.

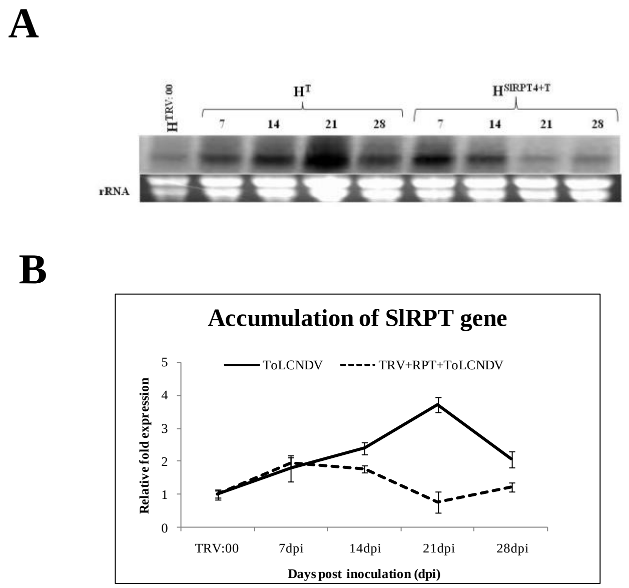

Supplementary Figure 4. Accumulation of *SlRPT4* in H^{T} (ToLCNDV) and $H^{SlRPT4+T}$ (TRV:SlRPT4+ToLCNDV). (A) Northern hybridization to evaluate the accumulation of *SlRPT* transcript. (B) Relative accumulation of *SlRPT4* in the experimental samples. Tomato plant infected with TRV: 00 vector was used as negative control. Bars show standard deviations (±SD). Ethidium bromide-stained total RNA has been shown as the equivalent loading control of the experiment.

A

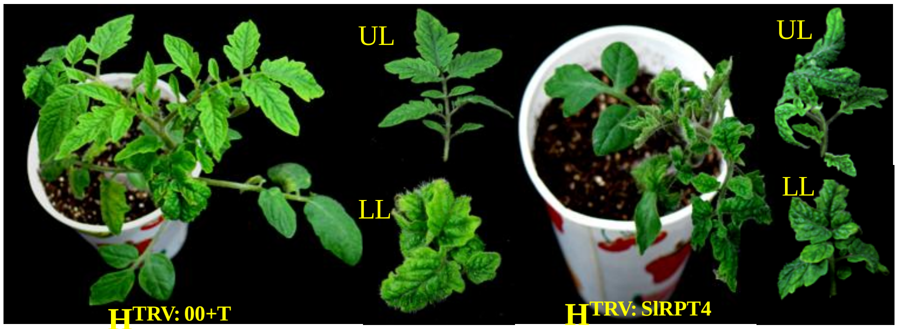

B

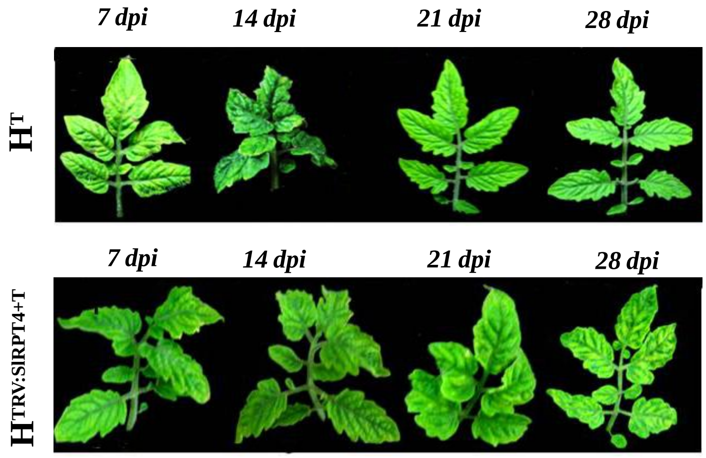

Supplementary Figure 5. Phenotype of Mock and SlRPT4 silenced cv. H-88-78-1 at 21 day post ToLCNDV infection. (A) Symptom remission. Systemic leaves showed symptom recovery in mock plants, however SlRPT4 silenced cv. H-88-78-1 was failed to recover from the ToLCNDV infection. $H^{TRV:00+T}$, mock plant infected with ToLCNDV; $H^{TRV:SlRPT4+T}$, SlRPT4 silenced plant infected with ToLCNDV, UL, upper leaf; LL, lower leaf. (B) Comparison of progression of leaf curl symptom between Control (H^{T}) and *SlRPT4* silenced cv. H-88-78-1 ($H^{TRV:SlRPT4+T}$) at 7-28 dpi of ToLCNDV.

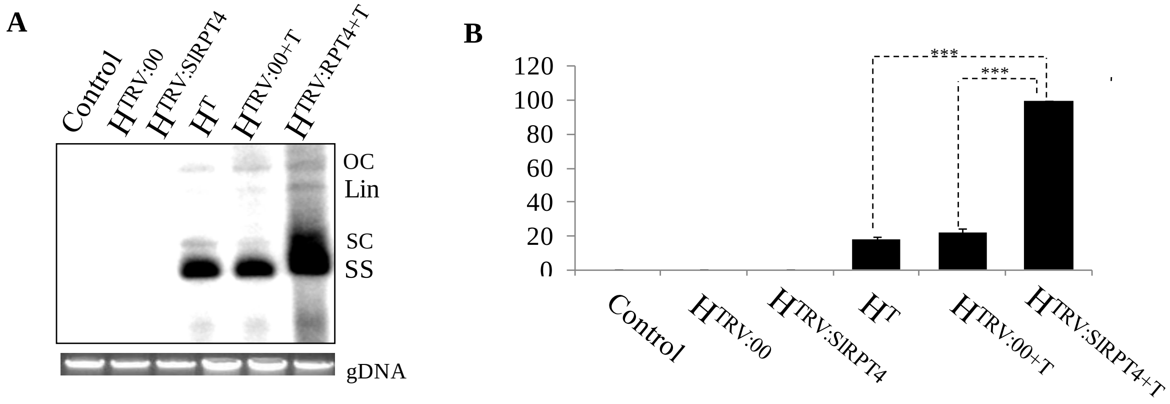

Supplementary Figure 6. Accumulation of DNA-B specific ToLCNDV molecule. Southern blot of tomato genomic DNA from all experimental plants were hybridized with ToLCNDV-BC1 (encoding Movement proteins) gene specific probe. Replicative forms of ToLCNDV genome are designate as open circular (OC), linear (Lin), supercoiled (SC) and single strand (SS). TRV:00 infiltrated H-88-78-1 was taken as a mock control. Ethidium bromide stained DNA from each experiments were shown as equivalent loading. (C) Relative accumulation of viral DNA in the samples HT and HSlRPT4+T at different time points. Data depicts means±SD of three independent experiments (n=3); *, P<0.05; **, P<0.01; ***, P<0.001.

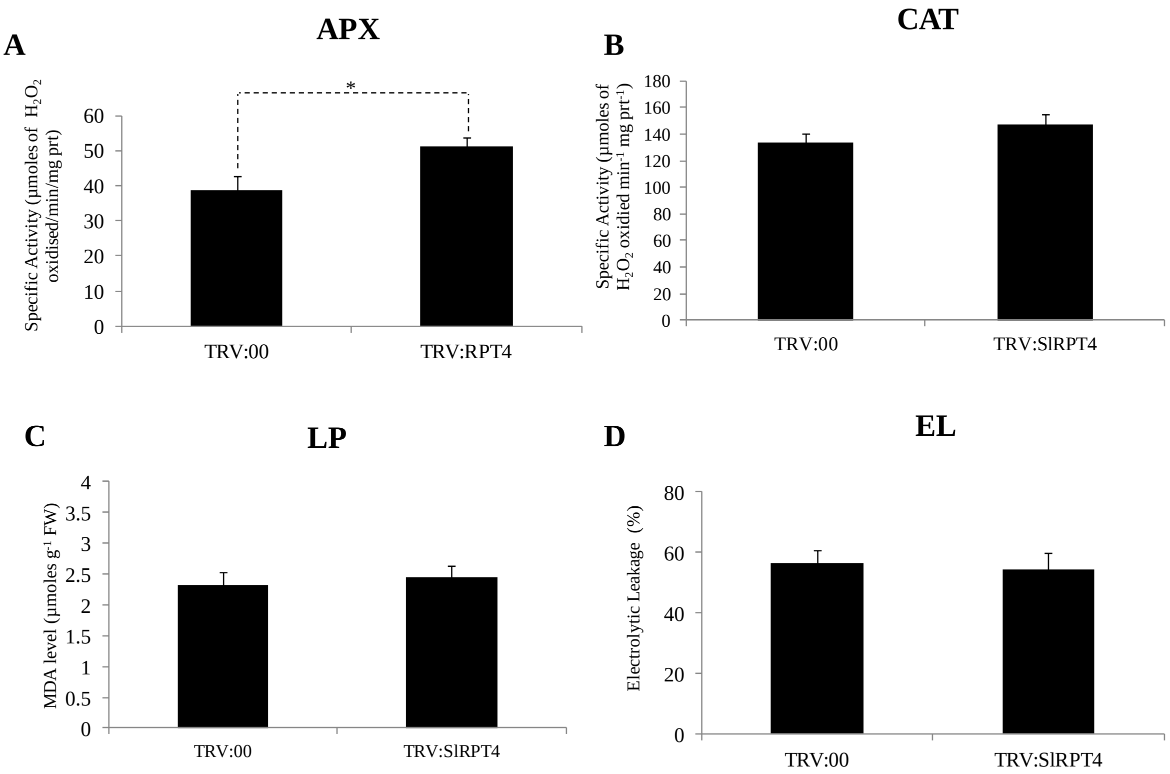

Supplementary Figure 7. Estimation of antioxidant enzyme activity in cv. H-88-78-1. (A) Specific activity of APX was measured as 1 µ mol of ascorbate oxidized min^{-1}. (B) Specific activity of CAT was measured as 1 µmol H_2O_2 oxidized min^{-1}. (C) Levels of lipid peroxidation expressed in terms of MDA concentration. (D) Percentage electrolytic leakage. Data depicts means±SD of three independent experiments (n=3); *, $P<0.05$; **, $P<0.01$; ***, $P<0.001$.Mock, TRV:00 infiltrated cv. H-88-78-1; TRV:SlRPT4; SlRPT4 silenced cv. H-88-78-1.

Supplementary Table S1. List of primers used in the study

**Primers used for bacterial expression of SlRPT4 protein**		
**Primer Name**	**Forward Primer Sequence**	**Reverse Primer Sequence**
pGEX4:RPT4	CGGGATCCATGGCGACCGAAGAAGACG	CGGAATTCTTATTCCTTGCCAAAATCAG
**Primers used for VIGS**		
pTRV-Slpds	CCGCTCGAGCTGACGAGCTTTCGATGCAGTGC	CGGGATCCATATATGGGACATTTATCACAGGA
pTRV-SlRPT4	CCGCTCGAGGTAATGCAGCCAACTCTTTCTCT	CGGGATCCCCTCTACTATATTACACCCCGTCCT
**Primers used for Southern blot analysis**		
Coat protein	ACAGAAAACCCAGAATGTACAGAA	CAACATTAAGGCATTTTCAGTATG
BC1	GTTTTGTGTCCCCCTCCGTCA	ATGTCAATAGGAAATGATGGTATG
**Primers used for transient expression analysis**		
pCAMBIA1302: SlRPT4	CATGCCATGGTAATGGCGACCGAAGACG	GGACTAGTTTATTCCTTGCCAAAATCAGCA
**Primers used for Northern blot analysis**		
Rep gene	TTTAAAGTGCTTTAGATAGTG	CACAATTACTTGTGTGGACAT
Coat Protein	ATGAAATTCACGCTACATGGCCTA	CGTTGAAATGATGATATCTGCTGG
SlRPT4	CCGCTCGAGGTAATGCAGCCAACTCTTTCTCT	CGGGATCCCCTCTACTATATTACACCCCGTCCT
Slpds	CCGCTCGAGCTGACGAGCTTTCGATGCAGTGC	CGGGATCCATATATGGGACATTTATCACAGGA
Nbpds	TAAACCCTGACGAGCTTTCGATGC	TTTATCACAGGAACTCCCACTAGC
αTubulin	TCAAACCTCAAAGAAGCTGTCA	ACAATTTATCCCTCACCACAGG
**Primers used for EMSA and ChIP assay**		
DNA-A-IR_EMSA (2592-47)	AAAACTTGTCGTTTTGATT	TGGTTGAGGGCCCACCTAAA
DNA-B-IR_EMSA (2617-67)	ACACCATATGGCATTATTGTAAT	AACGGCGTGCAATGATTACGC
DNA-A-Rep_EMSA (1939-2046)	GACTATGCTTATGGGCCTAAA	CCATTTTCAATTTCATCCT
IR_ChIP (2592-47)	AAAACTTGTCGTTTTGATT	TGGTTGAGGGCCCACCTAAA
pENTER-RPT4	CACCATGGCGACCGAAGAAGACGCCG	TTCCTTGCCAAAATCAGCACTGTAG
pENTER-RNA Pol-II-3	CACCATGGAGGGCGTTTCGTACCAG	TTAACCTCCACGCATATGAGCGCCCA
Actin 7	CTGGTGTGTGATAATGGAACG	GCTTCATCACCAACATACGC
